# Response to “Co-Culture Shock” by Behan & Mittelman: Co-culture shock? Let’s integrate!

**DOI:** 10.1038/s41420-025-02919-6

**Published:** 2026-01-27

**Authors:** Julia Zinngrebe, Pamela Fischer-Posovszky

**Affiliations:** 1 Department of Pediatrics and Adolescent Medicine, Ulm University Medical Center, Ulm, Germany; 2 German Center for Child and Adolescent Health (DZKJ), Partner site Ulm, Ulm, Germany

**Keywords:** Acute lymphocytic leukaemia, Cancer microenvironment

**TO THE EDITOR:**

We thank Drs. Behan and Mittelman for their interest in our recent publication “Bridging the marrow: a co-culture-platform of leukemia cells and MS5-derived stromal cells or adipocytes” [1]. We greatly appreciate their opinion and the extensive body of work they and others have contributed to understanding adipocyte-leukemia interactions via direct and indirect co-culture experiments [2–10].

No single model system perfectly recapitulates the in-vivo microenvironment of leukemia cells in the bone marrow, as also Drs. Behan and Mittelman emphasize. Originally, we set out to establish an easy-to-use and flexible co-culture platform with as little potential confounders as possible to study the interaction of fibroblasts and adipocytes with leukemia cells. This was a challenging task as adipogenic factors can influence survival and proliferation of leukemia cells. Most prominently and as expected, glucocorticoids had a massive impact on leukemia cell viability and proliferation (see Fig. 2 A, B in ref. [1]). We expected to obtain similar results with rosiglitazone as it was found to decrease acute lymphoblastic leukemia (ALL) cell numbers [11]. Insulin on the other hand is known to activate pro-survival pathways in leukemic cells [11] and might therefore induce ALL cell proliferation. Based on our findings, we suggest to remove adipogenic factors from the culture system by simply washing the cells prior to co-culture. This helps to exclude any differential and hard to control confounding influences of adipogenic factors on leukemic cells.

Stromal cells/adipocytes are adherent cells while leukemia cells grow in suspension. Each cell type requires specific media conditions which we carefully controlled for in our experiments. The effect of withdrawal of adipogenic medium II on MS5 adipocytes was assessed by us in Figure 3 [1] where we show that despite this withdrawal the cells still express comparable levels of adipogenic marker genes for up to 10 days after medium change. We also directly compared the influence of DMEM/F12 and alpha-MEM media on survival and proliferation of leukemia cells in co-culture with MS5 cells. Here, we did not detect any substantial differences in survival and only slight changes in proliferation (see Figure S6 in [1]). This indicates that the assay system per se is robust enough to tolerate different media conditions.

Our data obtained with B cell precursor (BCP)-ALL patient-derived xenograft (PDX) samples (Figure 5 A – C in [1]) in co-culture with MS5-derived stromal cells and adipocytes closely reflects findings from previously published in-vivo experiments in which leukemia cells proliferated poorly in adipocyte-rich environments [3, 5]. Our study did not aim to investigate the underlying reasons for the reduced efficacy of anti-leukemic therapy in patients with obesity [4, 6–8]. But as part of the establishment, we used our co-culture platform to assess the influence of adipocytes versus stromal cells on leukemia cell chemosensitivity. We were surprised that we could not reproduce previously published findings with our platform [2–10]. Possible explanations include differences in the tissue of origin of the stromal cells/adipocytes, the media composition and experimental readouts, or even a combination of those factors.

Although most of the studies used FCS in their co-cultures [3, 5, 6, 8], we think that the use of FCS should be minimized or at least carefully controlled for in co-culture experiments. FCS may mask supportive effects of feeder cells, especially when working with leukemia cell lines which do not depend on niche support for survival (see Figure 4 C in [1]). Although the growth factors and other components in the FCS may better mimic the in-vivo microenvironment of ALL cells as suggested by Behan and Mittelman, we would like to emphasize that FCS does not only support leukemia growth but also induces proliferation of the feeder cells. Thus, they can easily overgrow when cultured for prolonged periods of time. Irradiation can be used to induce a permanent growth arrest in feeder cells [2, 4, 7], but it may also trigger other unwanted alterations in the cells such as senescence [12]. Cellular senescence has been reported to enhance tumor cell proliferation, invasiveness, and resistance to therapy [13] and might therefore also affect the outcome of co-culture studies.

Behan and Mittelman mention that previous studies have used investigation periods of 24–72 h [2–8]. We have chosen 96 h and 7 days. We do not believe that this difference in culture duration is likely to account for the observed differences in chemosensitivity of leukemia cells in co-culture with stromal cells or adipocytes. It is unclear why any protective effect of adipocytes would be apparent only between 24 and 72 h but not at later time points. Importantly, untreated controls were always included in our assays and showed robust survival (Figure S8 C, E, G [1]), indicating sufficient nutrient availability during the course of the experiment. Additionally, the choice of experimental readout used to assess leukemia cell viability and proliferation may critically influence the results. Whereas previous studies counted surviving cells manually or with automated counters [2, 4, 6, 9], our study employed flow cytometry to assess viability and proliferation of leukemia cells using cell death and proliferation dyes following whole-well collection [1].

Finally, adipocytes in the bone marrow differ from adipocytes in white adipose tissue (WAT) in their gene expression and lipid composition [14]. In our study, we directly compared survival and proliferation of BCP-ALL cells (both cell lines and PDX samples) on bone marrow-derived MS5 to 3T3-L1 cells, which are a well-established model system of WAT biology (Figure S5 in ref. [1]). In our hands, MS5 cells were superior to 3T3-L1 cells in maintaining survival and proliferation of leukemia cells. Future studies will be required to clarify whether bone marrow and WAT-derived stromal cells/adipocytes exert differential effects on leukemia cell biology.

We thank Drs. Behan and Mittelman for their important thoughts on our manuscript. With our work, we aim to raise awareness that culture conditions can critically impact experimental results. We recognize that the serum- and adipogenic factor-free media conditions may have introduced additional stressors that could contribute to the observed sensitization of leukemia cells to chemotherapies in presence of adipocytes as compared to stromal cells. We hope that our findings will stimulate further research to deepen our mechanistic understanding of leukemia-adipocyte interactions by systematically comparing culture conditions in different model systems in vitro and ex vivo.
